# Crystal structure of 4-bromo-*N*-(2-hydroxy­phen­yl)benzamide

**DOI:** 10.1107/S1600536814024696

**Published:** 2014-11-15

**Authors:** Rodolfo Moreno-Fuquen, Vanessa Melo, Javier Ellena

**Affiliations:** a Departamento de Química – Facultad de Ciencias Naturales y Exactas, Universidad del Valle, Apartado 25360, Santiago de Cali, Colombia; b Instituto de Física de São Carlos, IFSC, Universidade de São Paulo, USP, São Carlos, SP, Brazil

**Keywords:** crystal structure, benzamide, hy­droxy­aniline, hydrogen bonding

## Abstract

In the title compound, C_13_H_10_BrNO_2_, the mean plane of the non-H atoms of the central amide C—N—C(=O)—C fragment (r.m.s. deviation = 0.004 Å) forms a dihedral angle of 73.97 (12)° with the hy­droxy-substituted benzene ring and 25.42 (19)° with the bromo-substituted benzene ring. The two aromatic rings are inclined to one another by 80.7 (2)°. In the crystal, mol­ecules are linked by O—H⋯O and N—H⋯O hydrogen bonds, forming chains along [010]. The chains are linked by weak C—H⋯O hydrogen bonds, forming sheets parallel to (100), and enclosing *R* ^3^_3_(17) and *R* ^3^_2_(9) ring motifs.

## Related literature   

For the anti­protozoal and anti­microbial properties of phenyl­benzamides, see: Ríos Martínez *et al.* (2014 ▶); Şener *et al.* (2000 ▶). For active metabolites of benzoxazoles, see: Mobinikhaledi *et al.* (2006 ▶). For studies of phenyl­benzamides as inhibitors of tyrosine kinases, see: Capdeville *et al.* (2002 ▶). For studies of phenyl­benzamides as inducers of apoptosis in biological processes, see: Olsson *et al.* (2002 ▶). For related structures, see: Fun *et al.* (2012 ▶); Hibbert *et al.* (1998 ▶).
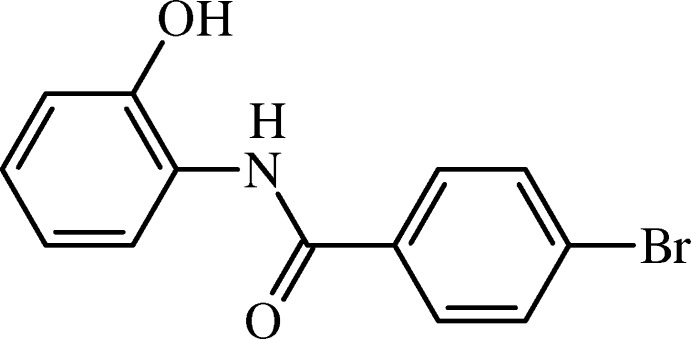


## Experimental   

### Crystal data  

C_13_H_10_BrNO_2_
*M* *_r_* = 292.13
Monoclinic, 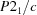

*a* = 23.4258 (10) Å
*b* = 5.6473 (1) Å
*c* = 9.2464 (3) Å
β = 93.008 (1)°
*V* = 1221.54 (7) Å^3^
*Z* = 4
Mo *K*α radiation
μ = 3.35 mm^−1^
*T* = 295 K
0.20 × 0.18 × 0.13 mm

### Data collection  

Nonius KappaCCD diffractometer
Absorption correction: multi-scan (*SADABS*; Sheldrick, 1996 ▶) *T*_min_ = 0.537, *T*_max_ = 0.662
21458 measured reflections
2490 independent reflections
1664 reflections with *I* > 2σ(*I*)
*R*_int_ = 0.063

### Refinement  

*R*[*F* ^2^ > 2σ(*F* ^2^)] = 0.052
*wR*(*F* ^2^) = 0.168
*S* = 0.99
2490 reflections
154 parameters
H-atom parameters constrained
Δρ_max_ = 0.61 e Å^−3^
Δρ_min_ = −0.68 e Å^−3^

### 

Data collection: *COLLECT* (Nonius, 2000 ▶); cell refinement: *SCALEPACK* (Otwinowski & Minor, 1997 ▶); data reduction: *DENZO* (Otwinowski & Minor, 1997 ▶) and *SCALEPACK*; program(s) used to solve structure: *SHELXS97* (Sheldrick, 2008 ▶); program(s) used to refine structure: *SHELXL97* (Sheldrick, 2008 ▶); molecular graphics: *ORTEP-3 for Windows* (Farrugia, 2012 ▶) and *Mercury* (Macrae *et al.*, 2008 ▶); software used to prepare material for publication: *WinGX* (Farrugia, 2012 ▶).

## Supplementary Material

Crystal structure: contains datablock(s) I, global. DOI: 10.1107/S1600536814024696/su5019sup1.cif

Structure factors: contains datablock(s) I. DOI: 10.1107/S1600536814024696/su5019Isup2.hkl

Supporting information file. DOI: 10.1107/S1600536814024696/su5019Isup3.cml

**Fig. 1.**
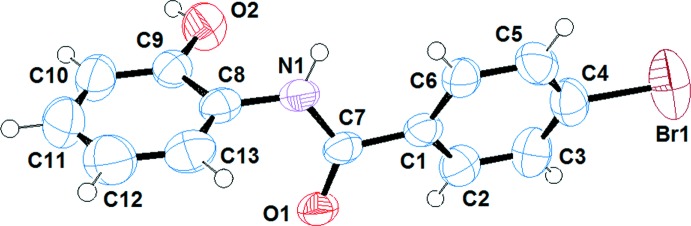
The molecular structure of the title compound (I), with atom labelling. Displacement ellipsoids are drawn at the 50% probability level.

**Fig. 2.**
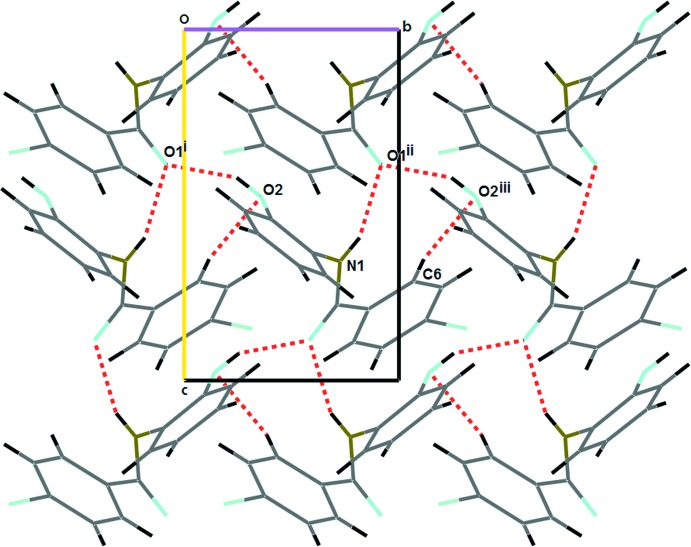
Part of the crystal packing of the title compound (I) viewed along the a axis, showing the formation of R33(17) and R32(9) ring motifs within the two-dimensional hydrogen bonded network running parallel to (100). Hydrogen bonds are shown as dashed lines; see Table 1 for details [symmetry codes: (i) x, -y-3/2, z-1/2; (ii) x, -y-1/2, z-1/2; (iii) x, y+1, z].

. DOI: 10.1107/S1600536814024696/su5019fig1.tif
The mol­ecular structure of the title compound (I), with atom labelling. Displacement ellipsoids are drawn at the 50% probability level.

a 3 3 3 2 . DOI: 10.1107/S1600536814024696/su5019fig2.tif
Part of the crystal packing of the title compound (I) viewed along the *a* axis, showing the formation of R^3^_3_(17) and R^3^_2_(9) ring motifs within the two-dimensional hydrogen bonded network running parallel to (100). Hydrogen bonds are shown as dashed lines; see Table 1 for details [symmetry codes: (i) x, −y-3/2, z-1/2; (ii) x, −y-1/2, z-1/2; (iii) x, y+1, z].

CCDC reference: 1033535

Additional supporting information:  crystallographic information; 3D view; checkCIF report

## Figures and Tables

**Table 1:** Hydrogen-bond geometry (, )

*D*H*A*	*D*H	H*A*	*D* *A*	*D*H*A*
O2H*O*2O1^i^	0.82	2.00	2.682(3)	141
N1H1O1^ii^	0.86	2.02	2.824(3)	155
C6H6O2^iii^	0.93	2.56	3.458(5)	164

Symmetry codes: (i) 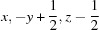; (ii) 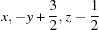; (iii) 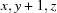.

## supplementary crystallographic information

### S1. Comment

The crystal structure determination of the title compound (I), is part of a study on phenylbenzamides carried out in our research group, and they are synthesized from the reaction of picryl benzoates with 2-hydroxy-aniline. These compounds have received extensive attention because of their antiprotozoal (Ríos Martínez *et al.*, 2014) and anti-microbialactivity (Şener *et al.*, 2000), and as active metabolites of benzoxazoles (Mobinikhaledi *et al.*, 2006). They have also been studied as inhibitors of tyrosine kinases (Capdeville *et al.*, 2002) and inducers of apoptosis in the tumor development process (Olsson *et al.*, 2002). Similar compounds to (I) have been reported in the literature, *viz.* 4-bromo-*N*-phenylbenzamide (II) (Fun *et al.*, 2012) and 2-hydroxy-*N*-benzoylaniline (III) (Hibbert *et al.*, 1998).

The molecular structure of (I) is shown in Fig. 1. The central amide moiety, C8—N1-C7(═O1)—C1, is essentially planar (r.m.s. deviation for all non-H atoms = 0.0026 Å) and it forms dihedral angles of 73.97 (12)° with the hydroxy-substituted phenyl ring and 25.42 (19)° with the bromo-substituted benzene ring. The bond lengths and angles within the molecule of (I) are in a good agreement with those found in the related compounds (II) and (III), although the N1-C7 bond length in the central amide segment, is slightly increased in structure (II), [C1-C7= 1.361 (2)Å].

In the crystal of (I), molecules are linked by O-H···O and N-H···O hydrogen bonds of medium-strength and weak C-H···O intermolecular contacts forming sheets parallel to (100) (Table 1 and Fig. 2). The O2-HO2···O1 hydrogen bonds are responsible for crystal growth in the *b* direction. In this interaction, the hydroxy O2-HO2 group in the molecule at (x, y, z) acts as a hydrogen-bond donor to atom O1 of the carbonyl group at (x ,-y-3/2, z-1/2). In turn, the N1-H1···O1 hydrogen bonds and weak C6-H6···O2 interactions, complement crystal growth in the *c* direction (see Fig. 2). The N1-H1 group of the amide moiety in the molecule at (x ,y, z) acts as hydrogen bond donor to carbonyl atom O1 in the molecule at (x, -y-1/2, z-1/2) and the C6-H6 group in the molecule at (x, y, z) acts as a hydrogen bond donor to atom O2 in the molecule at (x, y+1 ,z). Very likely, these interactions are responsible for the twist of the rings with respect to the central amide moiety. The combination of these interactions generate edge-fused R^3^_3_(17) and R^3^_2_(9) ring motifs.

### S2. Experimental

4-bromobenzoate 2,4,6-trinitrophenyl (0.050 g, 0.117 mmol) and 2-hydroxyaniline (0.0254 g) in molar ratio 1:2, were dissolved in 15 mL of toluene and mixed for 6 h under reflux and constant stirring. On completion of the reaction part of the solvent was evaporated and a crystalline black solid was obtained. [m.p.: 454 (1) K].

### S3. Refinement

The H-atoms were positioned in geometrically idealized positions and treated as riding atoms: O—H = 0.82 Å, N—H = 0.86 Å and C—H = 0.93 Å, with *U*_iso_(H) = 1.5*U*_eq_(O) for the hydroxyl H atom and = 1.2U_eq_(N, C) for other H atoms.

### Crystal data

**Table:**

C_13_H_10_BrNO_2_	*F*(000) = 584
*M**_r_* = 292.13	*D*_x_ = 1.588 Mg m^−^^3^
Monoclinic, *P*2_1_/*c*	Melting point: 454(1) K
Hall symbol: -P 2ybc	Mo *K*α radiation, λ = 0.71073 Å
*a* = 23.4258 (10) Å	Cell parameters from 2490 reflections
*b* = 5.6473 (1) Å	θ = 3.5–26.4°
*c* = 9.2464 (3) Å	µ = 3.35 mm^−^^1^
β = 93.008 (1)°	*T* = 295 K
*V* = 1221.54 (7) Å^3^	Block, black
*Z* = 4	0.20 × 0.18 × 0.13 mm

### Data collection

**Table:**

Nonius KappaCCD diffractometer	2490 independent reflections
Radiation source: fine-focus sealed tube	1664 reflections with *I* > 2σ(*I*)
Graphite monochromator	*R*_int_ = 0.063
CCD rotation images, thick slices scans	θ_max_ = 26.4°, θ_min_ = 3.5°
Absorption correction: multi-scan (*SADABS*; Sheldrick, 1996)	*h* = −29→29
*T*_min_ = 0.537, *T*_max_ = 0.662	*k* = −7→6
21458 measured reflections	*l* = −11→11

### Refinement

**Table:**

Refinement on *F*^2^	Primary atom site location: structure-invariant direct methods
Least-squares matrix: full	Secondary atom site location: difference Fourier map
*R*[*F*^2^ > 2σ(*F*^2^)] = 0.052	Hydrogen site location: inferred from neighbouring sites
*wR*(*F*^2^) = 0.168	H-atom parameters constrained
*S* = 0.99	*w* = 1/[σ^2^(*F*_o_^2^) + (0.0999*P*)^2^ + 0.5651*P*] where *P* = (*F*_o_^2^ + 2*F*_c_^2^)/3
2490 reflections	(Δ/σ)_max_ < 0.001
154 parameters	Δρ_max_ = 0.61 e Å^−^^3^
0 restraints	Δρ_min_ = −0.68 e Å^−^^3^

### Special details

**Table:**

Geometry. All esds (except the esd in the dihedral angle between two l.s. planes) are estimated using the full covariance matrix. The cell esds are taken into account individually in the estimation of esds in distances, angles and torsion angles; correlations between esds in cell parameters are only used when they are defined by crystal symmetry. An approximate (isotropic) treatment of cell esds is used for estimating esds involving l.s. planes.
Refinement. Refinement of *F*^2^ against ALL reflections. The weighted *R*-factor *wR* and goodness of fit *S* are based on *F*^2^, conventional *R*-factors *R* are based on *F*, with *F* set to zero for negative *F*^2^. The threshold expression of *F*^2^ > σ(*F*^2^) is used only for calculating *R*-factors(gt) *etc*. and is not relevant to the choice of reflections for refinement. *R*-factors based on *F*^2^ are statistically about twice as large as those based on *F*, and *R*- factors based on ALL data will be even larger.

### Fractional atomic coordinates and isotropic or equivalent isotropic displacement parameters (Å^2^)

**Table:**

	*x*	*y*	*z*	*U*_iso_*/*U*_eq_	
Br1	0.46192 (2)	1.31657 (9)	0.85256 (8)	0.1081 (3)	
O1	0.23094 (11)	0.5820 (4)	0.8853 (2)	0.0546 (6)	
C7	0.24418 (16)	0.7116 (6)	0.7843 (3)	0.0462 (8)	
C9	0.15972 (16)	0.4064 (6)	0.5411 (4)	0.0513 (8)	
O2	0.20914 (13)	0.3579 (5)	0.4767 (3)	0.0700 (8)	
HO2	0.2040	0.2472	0.4201	0.105*	
N1	0.21228 (13)	0.7256 (5)	0.6607 (3)	0.0520 (7)	
H1	0.2235	0.8168	0.5933	0.062*	
C1	0.29740 (16)	0.8587 (6)	0.7961 (3)	0.0490 (8)	
C6	0.30259 (17)	1.0664 (6)	0.7177 (4)	0.0565 (9)	
H6	0.2728	1.1161	0.6543	0.068*	
C8	0.16058 (15)	0.5964 (6)	0.6357 (3)	0.0500 (8)	
C5	0.3522 (2)	1.1998 (7)	0.7340 (5)	0.0675 (11)	
H5	0.3560	1.3385	0.6811	0.081*	
C2	0.34181 (18)	0.7886 (7)	0.8900 (4)	0.0604 (9)	
H2	0.3384	0.6499	0.9431	0.073*	
C4	0.39549 (18)	1.1262 (7)	0.8281 (5)	0.0665 (10)	
C13	0.1110 (2)	0.6596 (7)	0.7011 (4)	0.0683 (11)	
H13	0.1113	0.7876	0.7645	0.082*	
C10	0.11003 (19)	0.2786 (8)	0.5152 (5)	0.0696 (11)	
H10	0.1096	0.1490	0.4532	0.083*	
C11	0.0610 (2)	0.3429 (9)	0.5812 (5)	0.0816 (13)	
H11	0.0275	0.2566	0.5635	0.098*	
C3	0.39140 (18)	0.9208 (7)	0.9065 (5)	0.0704 (11)	
H3	0.4214	0.8717	0.9694	0.084*	
C12	0.06136 (19)	0.5340 (10)	0.6729 (5)	0.0851 (14)	
H12	0.0280	0.5786	0.7160	0.102*	

### Atomic displacement parameters (Å^2^)

**Table:**

	*U*^11^	*U*^22^	*U*^33^	*U*^12^	*U*^13^	*U*^23^
Br1	0.0702 (4)	0.0749 (4)	0.1796 (7)	−0.0166 (2)	0.0112 (4)	−0.0221 (3)
O1	0.0774 (17)	0.0477 (13)	0.0385 (12)	−0.0068 (12)	0.0028 (11)	0.0028 (10)
C7	0.062 (2)	0.0424 (17)	0.0345 (16)	0.0026 (15)	0.0049 (15)	−0.0055 (13)
C9	0.059 (2)	0.0450 (18)	0.0493 (18)	0.0008 (16)	−0.0007 (16)	0.0039 (15)
O2	0.0772 (19)	0.0527 (14)	0.0812 (18)	0.0004 (13)	0.0136 (15)	−0.0171 (13)
N1	0.071 (2)	0.0511 (15)	0.0340 (14)	−0.0087 (14)	0.0044 (13)	0.0005 (12)
C1	0.064 (2)	0.0451 (17)	0.0381 (16)	−0.0018 (15)	0.0080 (15)	−0.0056 (14)
C6	0.072 (2)	0.0474 (19)	0.0498 (19)	−0.0078 (17)	−0.0011 (17)	−0.0017 (15)
C8	0.061 (2)	0.0484 (18)	0.0405 (16)	0.0038 (16)	−0.0009 (15)	0.0046 (14)
C5	0.088 (3)	0.048 (2)	0.068 (2)	−0.0111 (19)	0.019 (2)	−0.0034 (17)
C2	0.069 (2)	0.052 (2)	0.060 (2)	0.0022 (18)	−0.0012 (19)	−0.0004 (16)
C4	0.062 (2)	0.056 (2)	0.082 (3)	−0.0074 (19)	0.012 (2)	−0.014 (2)
C13	0.075 (3)	0.075 (3)	0.056 (2)	0.010 (2)	0.0069 (19)	−0.0066 (18)
C10	0.072 (3)	0.065 (2)	0.070 (2)	−0.011 (2)	−0.007 (2)	−0.005 (2)
C11	0.062 (3)	0.099 (4)	0.081 (3)	−0.015 (2)	−0.013 (2)	0.007 (3)
C3	0.062 (2)	0.062 (2)	0.086 (3)	0.001 (2)	−0.004 (2)	−0.013 (2)
C12	0.056 (3)	0.119 (4)	0.081 (3)	0.010 (3)	0.005 (2)	0.005 (3)

### Geometric parameters (Å, º)

**Table:**

Br1—C4	1.895 (4)	C8—C13	1.384 (5)
O1—C7	1.239 (4)	C5—C4	1.365 (7)
C7—N1	1.334 (4)	C5—H5	0.9300
C7—C1	1.497 (5)	C2—C3	1.383 (6)
C9—O2	1.357 (4)	C2—H2	0.9300
C9—C10	1.380 (5)	C4—C3	1.374 (6)
C9—C8	1.384 (5)	C13—C12	1.375 (7)
O2—HO2	0.8200	C13—H13	0.9300
N1—C8	1.423 (5)	C10—C11	1.378 (7)
N1—H1	0.8600	C10—H10	0.9300
C1—C2	1.377 (5)	C11—C12	1.372 (7)
C1—C6	1.388 (5)	C11—H11	0.9300
C6—C5	1.387 (6)	C3—H3	0.9300
C6—H6	0.9300	C12—H12	0.9300
			
O1—C7—N1	122.0 (3)	C1—C2—C3	121.1 (4)
O1—C7—C1	120.9 (3)	C1—C2—H2	119.4
N1—C7—C1	117.2 (3)	C3—C2—H2	119.4
O2—C9—C10	123.4 (3)	C5—C4—C3	121.6 (4)
O2—C9—C8	116.7 (3)	C5—C4—Br1	118.7 (3)
C10—C9—C8	119.9 (4)	C3—C4—Br1	119.7 (3)
C9—O2—HO2	109.5	C12—C13—C8	120.3 (4)
C7—N1—C8	122.9 (3)	C12—C13—H13	119.9
C7—N1—H1	118.6	C8—C13—H13	119.9
C8—N1—H1	118.6	C11—C10—C9	120.1 (4)
C2—C1—C6	119.2 (3)	C11—C10—H10	120.0
C2—C1—C7	119.0 (3)	C9—C10—H10	120.0
C6—C1—C7	121.7 (3)	C12—C11—C10	120.2 (4)
C5—C6—C1	119.9 (4)	C12—C11—H11	119.9
C5—C6—H6	120.1	C10—C11—H11	119.9
C1—C6—H6	120.1	C4—C3—C2	118.6 (4)
C9—C8—C13	119.4 (4)	C4—C3—H3	120.7
C9—C8—N1	119.0 (3)	C2—C3—H3	120.7
C13—C8—N1	121.5 (3)	C11—C12—C13	120.1 (4)
C4—C5—C6	119.6 (4)	C11—C12—H12	120.0
C4—C5—H5	120.2	C13—C12—H12	120.0
C6—C5—H5	120.2		
			
O1—C7—N1—C8	0.8 (5)	C6—C1—C2—C3	−0.3 (5)
C1—C7—N1—C8	−179.6 (3)	C7—C1—C2—C3	−178.8 (3)
O1—C7—C1—C2	24.5 (5)	C6—C5—C4—C3	0.7 (6)
N1—C7—C1—C2	−155.1 (3)	C6—C5—C4—Br1	−178.2 (3)
O1—C7—C1—C6	−153.9 (3)	C9—C8—C13—C12	0.3 (6)
N1—C7—C1—C6	26.5 (4)	N1—C8—C13—C12	178.8 (4)
C2—C1—C6—C5	0.3 (5)	O2—C9—C10—C11	−178.0 (4)
C7—C1—C6—C5	178.7 (3)	C8—C9—C10—C11	1.3 (6)
O2—C9—C8—C13	177.9 (3)	C9—C10—C11—C12	−0.1 (7)
C10—C9—C8—C13	−1.4 (5)	C5—C4—C3—C2	−0.8 (6)
O2—C9—C8—N1	−0.7 (4)	Br1—C4—C3—C2	178.1 (3)
C10—C9—C8—N1	180.0 (3)	C1—C2—C3—C4	0.6 (6)
C7—N1—C8—C9	−107.5 (4)	C10—C11—C12—C13	−1.1 (7)
C7—N1—C8—C13	73.9 (5)	C8—C13—C12—C11	1.0 (7)
C1—C6—C5—C4	−0.5 (6)		

### Hydrogen-bond geometry (Å, º)

**Table:**

*D*—H···*A*	*D*—H	H···*A*	*D*···*A*	*D*—H···*A*
O2—H*O*2···O1^i^	0.82	2.00	2.682 (3)	141
N1—H1···O1^ii^	0.86	2.02	2.824 (3)	155
C6—H6···O2^iii^	0.93	2.56	3.458 (5)	164

Symmetry codes: (i) *x*, −*y*+1/2, *z*−1/2; (ii) *x*, −*y*+3/2, *z*−1/2; (iii) *x*, *y*+1, *z*.

## References

[bb1] Capdeville, R., Buchdunger, E., Zimmermann, J. & Matter, A. (2002). *Nat. Rev. Drug Disc.* **1**, 493–502. 10.1038/nrd839 12120256

[bb2] Farrugia, L. J. (2012). *J. Appl. Cryst.* **45**, 849–854.

[bb3] Fun, H.-K., Chantrapromma, S., Sripet, W., Ruanwas, P. & Boonnak, N. (2012). *Acta Cryst.* E**68**, o1269–o1270. 10.1107/S1600536812013487 PMC3344194 22606197

[bb4] Hibbert, F., Mills, J. F., Nyburg, S. C. & Parkins, A. W. (1998). *J. Chem. Soc. Perkin Trans. 2*, pp. 628–634.

[bb5] Macrae, C. F., Bruno, I. J., Chisholm, J. A., Edgington, P. R., McCabe, P., Pidcock, E., Rodriguez-Monge, L., Taylor, R., van de Streek, J. & Wood, P. A. (2008). *J. Appl. Cryst.* **41**, 466–470.

[bb6] Mobinikhaledi, A., Forughifar, N., Shariatzadeh, S. M. & Fallah, M. (2006). *Heterocycl. Commun.* **12**, 427-430.

[bb7] Nonius (2000). *COLLECT*. Nonius BV, Delft, The Netherlands.

[bb8] Olsson, A. R., Lindgren, H., Pero, R. W. & Leanderson, T. (2002). *Br. J. Cancer*, **86**, 97-1-978. 10.1038/sj.bjc.6600136 PMC2364155 11953831

[bb9] Otwinowski, Z. & Minor, W. (1997). *Methods in Enzymology*, Vol. 276, *Macromolecular Crystallography*, Part A, edited by C. W. Carter Jr & R. M. Sweet, pp. 307–326. New York: Academic Press.

[bb10] Ríos Martínez, C. H., Lagartera, L., Kaiser, M. & Dardonville, C. (2014). *Eur. J. Med. Chem.* **81**, 481–491. 10.1016/j.ejmech.2014.04.083 24865793

[bb11] Şener, E. A., Bingöl, K. K., Ören, I., Arpacı, Ö. T., Yalçın, İ & Altanlar, N. (2000). *Farmaco*, **55**, 469–476. 10.1016/s0014-827x(00)00070-7 11204748

[bb12] Sheldrick, G. M. (1996). *SADABS*. University of Göttingen, Germany.

[bb13] Sheldrick, G. M. (2008). *Acta Cryst.* A**64**, 112–122. 10.1107/S0108767307043930 18156677

