# Supplementary figures and images for: Crystal structure of 4-bromo-N-(2-hydroxy­phen­yl)benzamide
Source: Acta Crystallogr Sect E Struct Rep Online. 2014 Nov 15;70(Pt 12):o1261–2. doi: 10.1107/S1600536814024696 (PMC4257397; doi:10.1107/S1600536814024696)

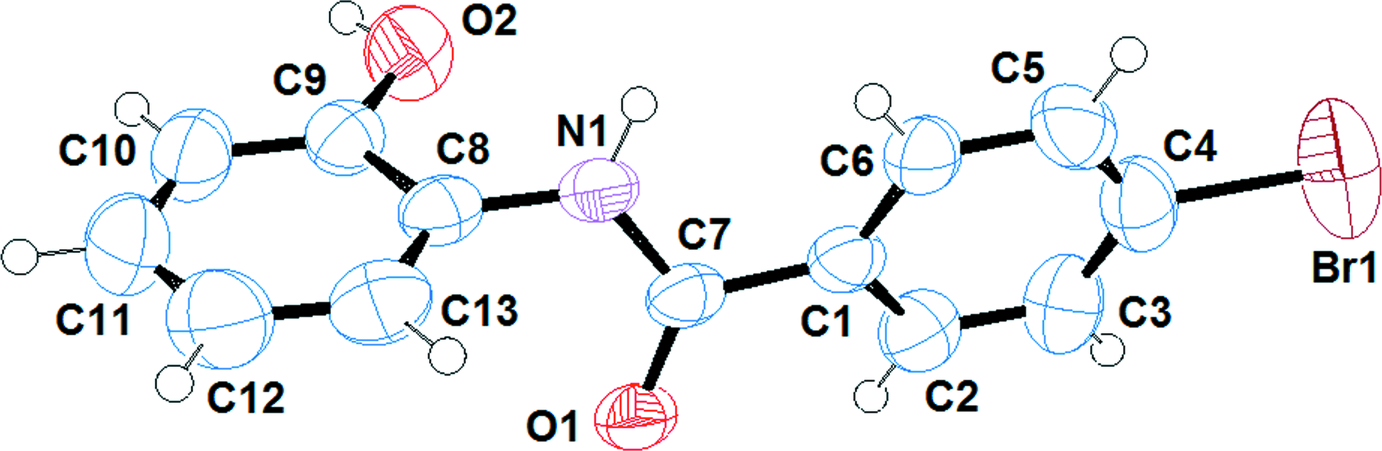

Supplement: Supplementary file 4 [file e-70-o1261-fig1.tif]

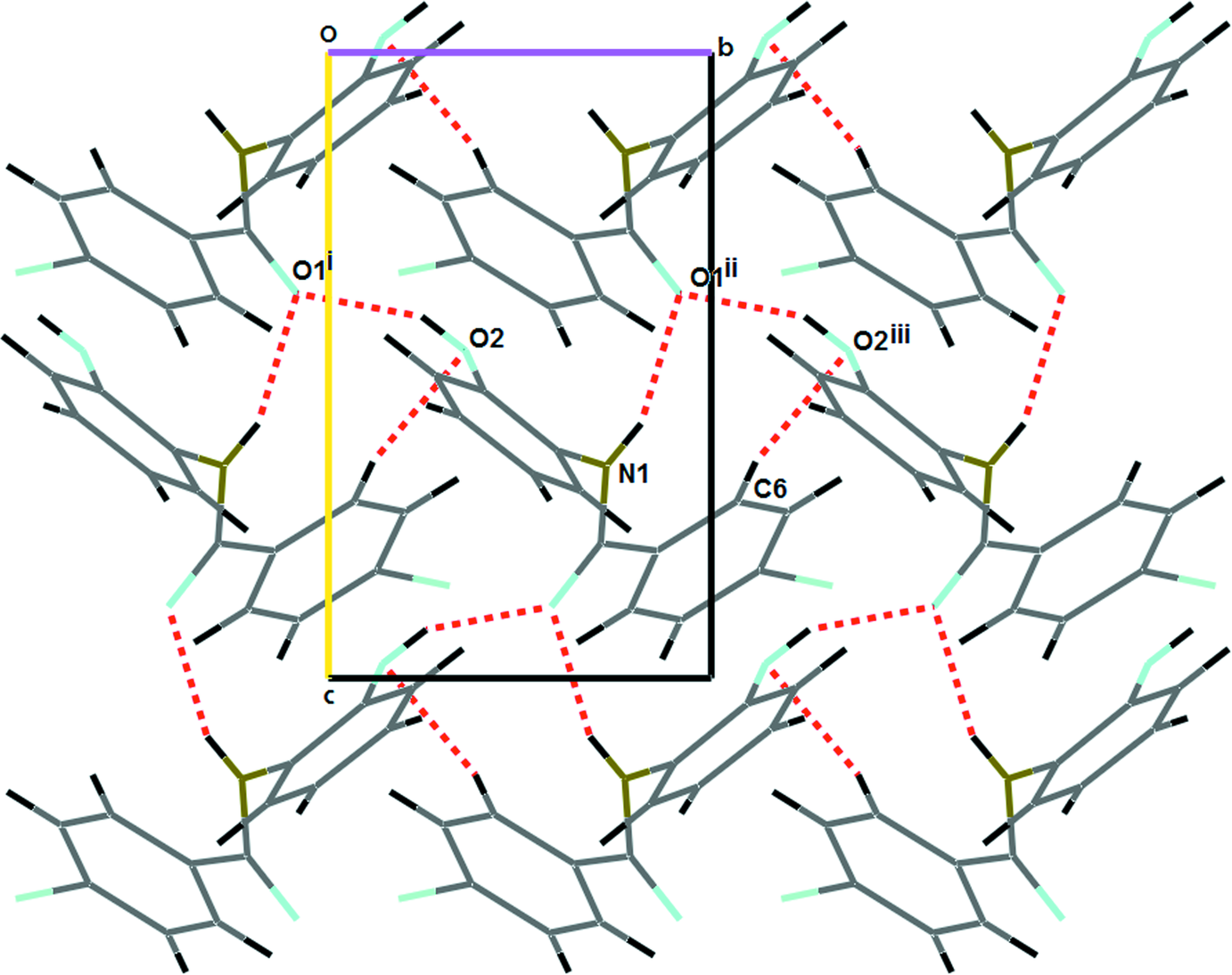

Supplement: Supplementary file 5 [file e-70-o1261-fig2.tif]
